# Partnerships in Learning: The Rural Health Multidisciplinary Training Programme in Aged Care Services Pilot Site Evaluation

**DOI:** 10.1111/ajr.70182

**Published:** 2026-04-04

**Authors:** Myles Clarkson Fletcher, Kathryn Fitzgerald, Mohammad Hamiduzzaman, Gordon Mander, Pascale Dettwiller, Lisa Dalton

**Affiliations:** ^1^ School of Nursing, UTAS Health University of Tasmania Hobart Tasmania Australia; ^2^ Western Australia Centre for Remote Health University of Western Australia Geraldton Western Australia Australia; ^3^ School of Allied Health, Australian Catholic University & School of Health Sciences The University of Sydney Camperdown New South Wales Australia; ^4^ Southern Queensland Rural Health University of Queensland Brisbane Queensland Australia; ^5^ Rural Health Academic, Centre for Rural Health University of South Australia Adelaide South Australia Australia; ^6^ Centre for Rural Health University of Tasmania Hobart Tasmania Australia

**Keywords:** aged care, clinical placements, interprofessional learning, qualitative evaluation, rural health education

## Abstract

**Objective:**

To explore the perspectives of University Departments of Rural Health (UDRH) clinical educators and partner aged care provider managers involved in the Rural Health Multidisciplinary Training (RHMT) Aged Care pilot programmes, and to identify the barriers, enablers and context‐specific strategies that supported the establishment of nursing and allied health student placements in rural and remote aged care settings.

**Setting:**

Five pilot sites were established through RHMT Aged Care Service Grants in partnership with aged care providers across New South Wales, Tasmania, South Australia, Western Australia and Queensland.

**Participants:**

Twelve participants, including nine UDRH clinical educators (CEs) and three partner aged care service managers, supported student placements at four of the five pilot sites.

**Design:**

Qualitative exploratory study using semi‐structured interviews. Data were analysed using a thematic approach.

**Results:**

Three interdependent themes were identified: (1) engaged partnership; (2) creating a learning environment that benefits everyone; and (3) adapting to the local context. Participants valued strong relational partnerships, role clarity and the CE role. While perceived benefits to students, services and staff ensured buy‐in to the value of creating a learning environment through placements. Feasibility required local adaptation to challenges, such as service capacity and workforce pressures.

**Conclusion:**

Rural aged care placements are feasible and valuable when supported by strong academic–provider partnerships, perceived benefits for everyone through establishing learning environments, and context‐sensitive implementation. These findings offer practical guidance for implementing aged care placements in rural settings, while highlighting the need for further research into student, staff, and resident outcomes.

## Key Points

**What is already known on this subject:**

- Rural aged care services face significant workforce shortages and limited access to student placements.
- Aged care placements are often perceived as low‐value by students and educators.
- Previous aged care programmes highlighted the importance of partnerships and educator roles but lacked sustained funding and did not target rural and remote services exclusively.

**What this study adds:**

- Rural aged care placements can be successfully established through relational academic–provider partnerships and dedicated clinical educator roles.
- Creating a mutual learning environment is important for implementation. This requires a shared belief that placements benefit staff and residents as well as students when successful.
- Local adaptation to challenges, while necessary for implementation, underscores the need for broader systems and structural changes, guided by appropriate research, if placements are to be scaled and sustained effectively.

## 1 Introduction

### 1.1 Background

An inverse care law persists in rural and remote aged care: services diminish as need increases with distance from metropolitan areas. Although rural older Australians make up a third of those aged over 65 years and a higher proportion of rural and remote populations, aged care services in these areas remain significantly fewer and less financially viable than in metropolitan centres [1, 2, 3]. Compared to their metropolitan counterparts, rural older Australians more often rely on lower‐level services, such as home support and home care [2], or have to move long distances to access residential care [4]. The availability of these services is constrained by workforce shortages and escalating costs in thin markets [3]. Rural demand for aged care services is projected to increase substantially in the coming years [5] and there is concern that delays in access will have cascading effects through to the health system [3].

Rural aged care providers face a dual challenge: attracting and retaining skilled workers amid the perceived lower status of aged care work [6] and overcoming the geographic and socioeconomic barriers to rural health workforce development [7]. Evidence indicates that supporting rural students to enter health courses and offering rural training opportunities can improve workforce recruitment and retention [8, 9, 10]. Similarly, aged care placement programmes benefit students, staff and residents when they have well‐resourced partnerships [11, 12, 13]. However, sustainability is limited and the sector continues to face high turnover and vacancy rates [14], and a projected workforce shortfall [15]. Although funded aged care places are increasing to meet demand, rural and remote services frequently lack capacity to deliver required care [3].

The employment landscape for allied health professionals in residential and community aged care settings remains uncertain. Despite the Royal Commission into Aged Care Quality and Safety recommending stronger allied health integration [16], recent reforms have primarily focused on regulatory and nursing standards rather than multidisciplinary staffing [17].

### 1.2 Policy Response

In 2020, the Commonwealth expanded the Rural Health Multidisciplinary Training (RHMT) Programme, establishing multidisciplinary aged care training sites through partnerships between University Departments of Rural Health (UDRHs) and aged care providers. Sites were required to establish governance arrangements, foster an education‐focused culture, and develop training spaces and resources for allied health and nursing placements. During the pilot phase (2022–2024), the five sites (Table 1) delivered 2358 placement weeks—35% nursing and 65% allied health students.

**TABLE 1:** Summary of five sites for the RHMT Aged Care Pilot programme 2022–24.

UDRH	Aged care partner	MMM	Placement opportunities
Southern Queensland Rural Health (SQRH)	Southern Cross Care Queensland's Illoura Village residential aged care facility in Chinchilla	5	Exercise physiology, nursing, dietetics, psychology, social work, pharmacy, speech pathology and occupational therapy
University of South Australia Department of Rural Health (SA DRH)	Matthew Flinders Aged Care Services, Port Lincoln SA	6	Nursing, physiotherapy, occupational therapy, speech pathology, pharmacy and social work
University Centre for Rural Health, University of Sydney (UCRH, UoS)	Whiddon Aged Care Homes (Kyogle and Casino)	4, 5	Physiotherapy, occupational therapy, social work, speech pathology, nutrition and dietetics
Centre for Rural Health (CRH), University of Tasmania	Corumbene, an independent not‐for‐profit aged and community care provider based in the Derwent Valley	2–5	Nursing, physiotherapy, speech pathology, paramedicine, pharmacy
Western Australia Centre for Rural Health (WACRH)	WA Country Health Service (WACHS) focussing on Carnarvon Health Campus—a multi‐purpose service site that includes Gnullingoo Mia Residential Care, inpatient, outpatient and community services	6	Nursing, physiotherapy, pharmacy, speech pathology, occupational therapy, dietetics, paramedicine, social work, public health, medical imaging

Each site undertook a localised co‐design process between UDRH staff, aged care managers, and regional stakeholders to determine feasible disciplines, supervision structures, and timelines, aligning with RHMT objectives.

### 1.3 Study Aims and Rationale

This study examined the perspectives of UDRH clinical educators (CEs) and aged care managers who participated in the initial phase of the RHMT Aged Care pilot programmes. Specifically, it aimed to identify barriers, enablers, and strategies underpinning successful placement establishment in rural and remote aged care.

While individual sites continue to report student activity data and site‐specific evaluations, this study provides a cross‐site analysis of the foundational work required to initiate and sustain aged care placements in rural contexts. By focusing on early implementation, it adds to the evidence base and provides practical insights for scaling such models. It also addresses a gap by examining relational and contextual factors underpinning placement feasibility in exclusively rural aged care settings, prior to assessing student outcomes and local impacts.

## 2 Methods

### 2.1 Ethics

An initial low risk application was submitted to the University of Queensland and approved (Approval ID 2023/HE002384). This was then ratified and approved by the University of Western Australia (Approval ID 2024/ET000126). Subsequent ethics submissions and approvals were made to the University of Tasmania (Approval ID H30133) and the University of South Australia (Approval ID 206218). The University of Sydney registered the initial University of Queensland ethics application.

All participants provided written informed consent, including permission for audio‐recording and de‐identified transcript sharing among research collaborators via a secure server.

### 2.2 Study Design

A qualitative‐exploratory design was used as the research strategy. This approach is best suited to descriptive examinations of a phenomenon within its real‐world context over time [18]. A series of semi‐structured interviews was conducted with UDRH CEs and aged care managers who had supported RHMT placements during the first 3 years. A hybrid inductive‐deductive analytic framework guided coding and theme development [19], integrating emergent concepts with relevant literature.

### 2.3 Settings and Participants

The study was conducted across the five RHMT Aged Care pilot sites, which included both residential and community‐based aged care services located in Modified Monash Model (MMM) 2–6 regions (NSW, QLD, SA, TAS, WA) (Table 1). Each aged care provider was partnered formally with a UDRH under grant funding to develop multidisciplinary placement capacity.

A purposive sampling strategy was used to recruit participants with direct involvement in the design, implementation, and facilitation of student placements at the five sites. All clinical educators and aged care managers who were involved in the early phase of the aged care pilots (2022–2024) were identified by the site leads and invited to be interviewed. Invitation emails, including study information and consent forms, were distributed by the project manager. Participants returned signed consent forms prior to scheduling interviews.

### 2.4 Research Team and Reflexivity

The research team comprised six members with expertise in rural health education, qualitative research and aged care work. The project manager was known to some UDRH staff but was not a line manager or supervisor and was not involved in the RHMT Aged Care pilot project implementation. Three researchers led the study design (G.M., K.F., M.C.F.), and interviews were conducted by researchers not employed by the participants' UDRH or aged care service to reduce bias. The analysis team included members with disciplinary backgrounds in nursing and allied health and experience in qualitative thematic analysis.

### 2.5 Data Collection

The semi‐structured interviews were conducted between 27 March and 15 August 2024 via Zoom. To ensure a consistent interview approach, a semi‐structured interview guide comprising 13 questions was developed and pilot‐tested within the team and by an external UDRH staff member (see Table 2). Interviews ranged between 25 and 40 min.

**TABLE 2:** Interview question guide—clinical educator.

No.	Transition question
1	Can you tell me a little about your job/position? Discipline Employment, for example UDRH, private, facility/organisation

	Key questions
	*Section 1: Background and experience*
2	Tell me how you became involved in this student supervision programme and your role with students. Prompts: Reason/how they became involved—nominated, required as part of role How long has involvement in the aged care training programme been Overall experience as a CS/P
	*Section 2: Nature of student placements*
3	Can you describe the student activities and the supervision that you provide in [this programme]? Prompts: What type of supervision—do they ○ coordinate placements, ○ are they the principal CE (AH) or preceptor (nursing), occasional supervisor Any supervision methods—debrief, peer assisted, observation etc. IPL or discipline‐specific (or both) Types and numbers/frequency of students supervised (over a period of time) in this programme.
4	Please tell me about the any preparation and any support provided as part of this aged care training programme—for you as the supervisor? Prompts: Training in supervision Additional time UDRH support/coordination Physical resources (e.g., IT, equipment for teaching, clinical resources) Staff—additional? (in organisation/UDRH)
5	What preparation are you aware of that students participate in for this placement—with you or others? Prompts: What does orientation look like (before or during placement) Preparation for working with aged care conditions, for example dementia, chronic conditions often seen in older population Any specific local requirements, for example CALD Preparation for living away from home?
	*Section 3: Challenges and impact*
6	Can you tell us what you think about how and if this placement prepares students to work in rural aged care in the future? Prompts: Types of experiences May need to separate rural and aged care
7	From your perspective as a supervisor/preceptor, how do the placements in this programme differ from other student placements? Prompts: Expanded or limited experiences Supervision Length Support, for example accommodation, UDRH
8	Do you feel having students in [facility name] has impacted the care of individual residents/clients? Prompts: Positive/negative Type or care Amount of care General impacts, for example interaction
9	Have you noticed any benefits or improvements in the aged care setting/[name of organisation] due to the presence of student placements? Prompts: activities services projects
10	Could you share any challenges or difficulties faced as a clinical supervisor/preceptor in [programme name]? Prompts: For the Student Supervisors Clients/residents Workplace UDRH How do you address these challenges?
	*Section 4: Suggestions for development/improvement*
11	Do you have any suggestions on how the aged care students' placements can be improved? Prompts: student numbers (more/less) breadth of student disciplines support for supervisors support from UDRH
12	As a clinical supervisor/preceptor, can you suggest any support or resources that would help you in that role?
13	Is there anything else about the student supervision, student activities or the [training programme name] that we haven't had a chance to talk about yet that you would like to?

All interviews were audio recorded and transcribed verbatim via a third‐party transcription service (SmartDocs, Lexical Ventures Pty Ltd) and participants were provided with a copy of the transcript for member checking. The deidentified transcripts were stored on a secure University of Queensland server and shared via a password‐protected link among authorised team members.

### 2.6 Analysis

Data were analysed using a flexible thematic approach [20] supported by NVivo (Lumivero). Coding followed four stages: familiarisation with the data; generating codes; grouping codes into overarching themes; reviewing and refining themes before presenting the findings [21]. A hybrid approach was used, in which both inductive (data‐driven coding) and deductive (theory‐informed) reasoning allowed for integrative examination of emergent concepts in relation to relevant literature [19, 22].

**FIGURE 1:**
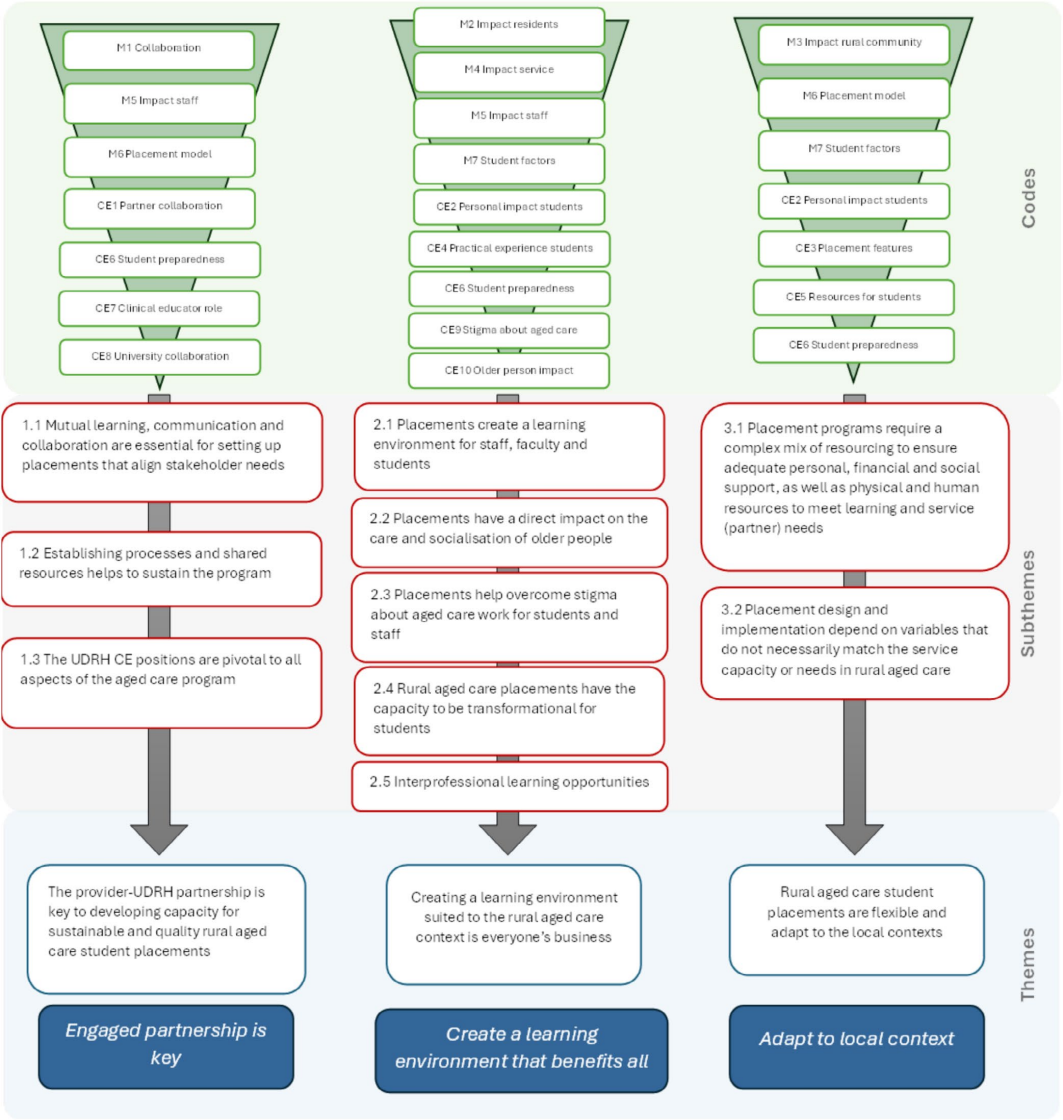
Data analysis codes, subthemes and themes.

Data were initially coded individually using a line‐by‐line inductive approach, involving three researchers (K.F., M.C.F., L.D.). Manager and CE interviews were coded separately, then compared to ensure coherence. The initial coding round was discussed by the research team for accuracy and agreement and further refining. The refined codes were organised into 10 subthemes (Figure 1).

Three overarching themes were derived from the subthemes and reviewed in reference to literature about rural and aged care student placements. All subtheme categories were checked for fit within the themes, which were then named and described [22]. The research team referred to the Consolidated Criteria for Reporting Qualitative Research (COREQ) guidelines [23] to maintain transparency for reporting.

## 3 Results

### 3.1 Semi‐Structured Interviews

Twelve interviews were conducted; three with managers of the aged care partners from three sites, and nine UDRH employed CEs from four of the sites. The CEs included two registered nurses and seven allied health professionals including physiotherapy, occupational therapy, exercise physiology and social work. There were no managers from two sites. One partner organisation did not approve ethics so no managers could be approached. Another site did not have an eligible manager from the pilot phase due to staff turnover.

### 3.2 Data Saturation

Data saturation was assessed retrospectively following Guest et al. [24]. Given the limit of the number of participants, this approach provided a pragmatic framework for assessing thematic completeness. A ≤ 5% new information threshold indicates a low rate of new information in later interviews [24].

While manager data alone did not meet the threshold because of small numbers, integration with CE data produced a pooled threshold of 4%, supporting confidence in theme completeness (see Table 3).^1^

**TABLE 3:** data saturation for semi‐structured interviews.

Manager interview	M1	M2	M3
New codes/interview	18	2	2

CE interview	CE1	CE2	CE3	CE4	CE5	CE6	CE7	CE8	CE9
New codes/interview	27	6	3	2	2	5	1	1	1

*Note:* Base size: (M1 + M2) 20; Run length: 1 (M3); New information threshold: 5%. Base size: (CE1–CE7) 46; Run length: 2; New information threshold: 4%.

#### 3.2.1 Theme 1: Engaged Partnership Is Key

Creating rural aged care student placements depended on actively tended partnerships between the UDRHs and the aged care providers. In settings with workforce instability and competing service pressures, relationship building was a continuous and essential focus.

So, I think one of the biggest challenges is residential aged care homes are really busy places, and so fitting in with an already existing inter‐professional team that's totally under the pump comes with challenges. So really having to build relationships at sites. And then of course you build with certain staff and then they're gone, so then you're re‐doing the process the whole time. (CE, 6)

Participants emphasised that partnerships must extend beyond executive agreements to day‐to‐day collaboration with the teams hosting students, ensuring the models worked on the ground as well as on paper.

[…] looking at the best ways to working in partnership with those sites, which I think we're doing, but I think sometimes maybe it's more on our terms and we need to try and really look at that as just continuing those collaborative partnerships. I think part of that is we've been doing a lot with executive teams, but then how we establish that with all of the team on the ground is probably the big thing we're trying to do. I think that's ongoing communication, I think it's about having key staff that can keep that going. (CE, 6)

Partnerships thrived when communication was routine and issues were resolved early, keeping all parties aligned on aims and expectations.

So, that has been key, that we've had a really good working group and sorting out those little humps and bumps along the way. And that's an ongoing relationship. (M, 2)

CEs were the connective tissue of the programme—linking universities, services and students—while creating psychological safety and tailoring activities to learners' needs.

I work in a student‐centred way… building psychological safety… then structuring activities from there. (CE, 6)

Many of the CEs were motivated by the opportunities the programme provided.

I'm really passionate – I grew up in the city and having worked, lived rurally and just seeing the difference that you can make in people's lives has really been a big motivation for me. (CE, 7)

Shared processes and resources—especially robust orientation and clarity about responsibilities—helped placements run smoothly despite staff turnover and busy care environments.

We needed strong processes… everyone knew whose role was what and who would orientate the student. (M, 2)

#### 3.2.2 Theme 2: Create a Learning Environment That Benefits Everyone

The participants observed how clinical placements were beginning to function as shared learning ecosystems, which they perceived as benefiting students, staff and residents.

Managers noted that students brought fresh eyes and best‐practice perspectives that prompted opportunities for staff reflection.

Having different students, not just from nursing, has been fantastic… it puts a different point of view on what we're doing. (M, 1)

And I think because our staff see the benefit for the residents, then that's making their roles easier as well. (M, 2)

Participants described perceived benefits for residents, including increased services through student placements and improved social and emotional wellbeing linked to greater interaction with students.

We've seen residents walk that haven't walked. We've seen residents get back into hobbies and things that they've enjoyed. We've got self‐reports of residents talking about how much their life is improved in the residents meeting. (CE, 7)

Of note, participants highlighted the benefits of establishing placements for allied health students from professions that traditionally lacked these services.

[…]we had exposure to physio, OT, speech ‐ and we have had one pharmacy student ‐ the services available to our residents [sic] has just dramatically increased. We've had lots and lots of improvements by having student‐led projects and student‐led clinical services. (M, 3)

For staff and students, these experiences were thought to challenge the stigma often associated with aged care work and revealed its potential for meaningful contribution.

I think that perceptions do change around what aged care actually is and what a profession or what a career in aged care could look like and the relationships they can build. (CE, 2)

I think it's really fantastic for our staff because it ‐ yeah, it just takes them back to, “Yeah, I'm here for a purpose”. (M, 3)

CEs felt that students had opportunities to develop their professional identity through their contribution to.

So, I think students see a gap and they go, ‘Oh, actually that person wouldn't have had anything if I wasn't here’. So, they leave feeling like they've contributed and done something really positive for the older people that they work with. (CE, 6)

Finally, placements were seen to foster interprofessional learning as students collaborated across disciplines in authentic team‐care contexts.

The students have consistently reported… that they have better interactions with students of other disciplines. It's not so much students crossing in the corridors on placement or having discussions at lunchtime, it's a bit more that they're able to collaborate, they're able to work closely together. (CE, 2)

#### 3.2.3 Theme 3: Adapt to the Local Context

While partnerships and mutual learning were identified as enablers for placement establishment, numerous challenges were also described, which required continual adaptation to the local workforce and service realities.

Service models and local workforce determined which disciplines could be hosted; misalignment between placement plans and on‐site capacity required flexible design.

If I was an OT CE in [location], we'd have an OT program… but there's no staff to deliver that service. (CE, 1)

Supervision capacity, particularly the availability of registered nurses (RNs), had to be balanced with learner needs and the constraints of small teams.

We were struggling with RN numbers… so it was about reassurance, support and education to bring staff on board. (M, 2)

Staff fatigue emerged as a consideration, especially early in implementation.

[…] in the very beginning, it was probably a bit of fatigue on our staff because of the extra time that's needed ‐ the students want to talk [to] the staff and want to get their input. So, it's a bit of a challenge in [sic] their fatigue. But we recognised that quickly, and the uni have been great and done a lot of student‐led education for them and some support. (M, 2)

Short placement rotations disrupted continuity for residents and staff; staggering and pairing strategies helped but did not fully remove the load on CEs.

Continuity can be a real challenge… residents and staff are always getting to know new people. (CE, 6)

However, dedicated training spaces and co‐located accommodation can amplify interprofessional interaction and ease collaboration.

Having all the students in one place […] has been great for interprofessional learning. (CE, 2)

Yet, physical and logistical constraints—limited training rooms, travel distances, and IT support—shaped what was feasible.

It would be lovely to have a bit more training space […] the travel is tricky. (CE, 7)

Across sites, adaptability emerged as both a constraint and enabler. Placements succeeded where models were tailored to the workforce, infrastructure, and community context.

## 4 Discussion

This qualitative exploration of the aged care pilot training sites demonstrates that rural aged care student placements are feasible and potentially valuable when supported by three interdependent conditions: engaged academic–provider partnerships, mutually beneficial learning environments and context‐sensitive adaptation. These findings are based on the perspectives of clinical educators (CEs) and aged care managers who were key stakeholders in the implementation of the pilot programmes. While the study does not include the perspectives of students, staff or residents, the insights offered provide a foundational understanding of the structural and relational work required to establish allied health and nursing student placements in rural aged care settings. These findings are consistent with evidence from effective student placement models in other rural health settings [25, 26, 27] and previous aged care programmes [12, 28, 29, 30, 31]. Both rural and aged care settings require effective collaboration with local providers and understanding of the realities of their work in order to establish relevant health student placements.

### 4.1 Engaged Partnership Is Key

Across the sites, CEs and managers described partnership work as foundational to initiating placements. Formal governance arrangements provided legitimacy, but it was the day‐to‐day collaboration (through role clarity, shared orientation processes, and routine communication) that enabled placements to function under real‐world pressures. The CE role emerged as the critical enabler within these partnerships, forging connections between universities, providers and staff, and students. Beyond functioning solely as placement coordinators, CEs undertook relational, organisational, and educational work that supported trust‐building, aligned expectations, and adapted placement activities to service realities. This finding aligns with broader rural placement literature, which highlights the importance of dedicated clinical education roles and sustained engagement to establish placement capacity in settings characterised by workforce instability and competing service demands [9, 25, 26, 32].

Comparable models emphasise the time‐intensive nature of partnership development. For instance, the Three Rivers Placement Model (TRPM) recommends a 12‐month process of stakeholder engagement to co‐design placements in rural and remote regions [25]. The work of the CEs in this study also reflects the principles of ‘community literacy’ as described by Jones et al. [33], where partnerships serve as conduits for mutual understanding between education and service sectors and their communities. Similarly, aged care placement programmes, such as Teaching and Research Aged Care Services (TRACS), highlight the time and relational investment required to bridge the cultural gap between the education and aged care sectors [28].

A recent systematic review of nursing placements in aged care [31] led to a best practice summary, recommending formal academic–provider partnerships and the assignment of dedicated faculty to create learning opportunities for students and staff [34]. Baptiste et al. [35], in their discursive review of approaches to academic–provider partnerships, emphasise the multifaceted role of facilitators in establishing effective collaborations.

This theme suggests that partnership is not a static condition but an ongoing process that underpins feasibility in rural aged care. The reliance on relational labour also highlights the vulnerability of placement models that depend on individual roles and local goodwill, reinforcing the need for structural support to sustain partnerships over time.

### 4.2 Create a Learning Environment That Benefits Everyone

Across the pilot sites, CEs and managers described placements as creating learning environments that appeared to benefit students, staff, and residents. Although this evaluation did not measure resident or service‐level outcomes, participants consistently reported *perceived* gains, including increased resident engagement, opportunities for staff reflection, and meaningful student contributions to care. Similar perceived benefits have been documented in other aged care placement programmes, where improvements in morale, practice reflection, and resident interaction were reported even in the absence of formal outcome metrics [12, 28, 29, 30, 31].

The literature suggests that positive staff and student experiences play a meaningful role in shaping the acceptability and ongoing viability of placements [27, 36]. Staff willingness to supervise and host students is strongly influenced by whether placements are *perceived* to add value rather than burden overall [26, 30, 31]. In the TRACS programme, shifts in staff perception of the impact of students were critical to embedding education models [28]. Likewise, rural placement research indicates that positive student experiences have been associated with stronger engagement in interprofessional learning, confidence, and consideration of future rural practice [25, 26, 27, 36].

Framing these findings as *perceived* mutual benefit reflects both the current evidence and the early development stage of the pilot sites. Such perceptions supported local buy‐in, reinforced the value of academic–provider partnerships, and contributed to the emergence of a learning culture, elements considered foundational for longer‐term embedding of placements [28, 29, 37]. However, these perceptions should be interpreted as early enablers rather than sufficient indicators of sustainability, which will depend on structural factors such as supervision capacity, workforce stability, and organisational support [38].

### 4.3 Adapt to the Local Context

The theme of local adaptation reflects the ways in which participants navigated structural challenges inherent to aged care [39] and rurality [9], including workforce shortages, limited discipline‐specific supervision, pressure on RN availability, staff fatigue, constrained training spaces, and logistical barriers such as travel and IT support. These challenges align with long‐standing structural issues in rural and remote aged care [5, 40, 41]. Such issues were not resolved by hosting students, but participant responses to them highlighted how adaptability became the mechanism that allowed sites to navigate them sufficiently to initiate and support placements.

Adaptations such as staggered student rotations, flexible supervision models, co‐located accommodation, and blended placement designs enabled sites to initiate and maintain placements within these constraints. Broader rural placement evidence demonstrates that flexible, context‐sensitive design is necessary for placements to function in environments characterised by workforce shortages, limited supervisory capacity, and high service demand [26, 27, 30, 31].

The need for adaptation can be seen to underscore, rather than diminish, the depth of the systemic challenges identified. While adaptability enabled short‐term solutions, sustainability will depend on addressing these conditions at a systems level, including workforce stability [42, 43, 44, 45], supervision capacity [31, 32], physical infrastructure limitations [28, 30], and organisational readiness for student involvement [29, 37].

Viewed in this way, placements functioned as both a training mechanism and a diagnostic lens, revealing gaps in service capacity and areas requiring longer‐term investment. Moving from adaptive coping to more system‐supported placement models will be essential if rural aged care placements are to contribute meaningully to workforce development and service sustainability.

### 4.4 Implications

This multi‐site evaluation indicates that the feasibility of establishing rural aged care student placements rests as much on the intrinsic value of placements as on the structural and relational conditions that support them. The findings underscore the importance of well‐resourced academic–provider partnerships, with dedicated clinical educator roles functioning as the primary mechanism for aligning educational objectives with service realities.

While *perceived* benefits for students, staff, and residents were important in generating early engagement and fostering a local culture of learning, these perceptions alone are insufficient to ensure placement sustainability. Durability depends on broader structural enablers, including supervision capacity, workforce stability, organisational readiness, and infrastructure support. For policy and programme development, these findings align with the intent of the RHMT programme and national aged care workforce strategies [9, 46], suggesting that expansion of placements into rural aged care settings should prioritise sustained investment in partnership structures and flexible, context‐sensitive implementation rather than uniform scaling.

### 4.5 Limitations and Future Research

In interpreting these implications, several limitations should be acknowledged. This study reflects the perspectives of CEs and managers during the early establishment phase and does not include the views of students, residents, or non‐managerial staff. Findings are based on *perceived*, rather than measured, outcomes and should be interpreted accordingly. While participants represented four of the five pilot sites, the qualitative design limits generalisability.

Future research should examine the measurable impacts of rural aged care placements on resident outcomes, service delivery, staff capability, and student preparedness for rural and aged care practice. Mixed‐methods and longitudinal multi‐site evaluations will be essential to understand how placements interact with broader structural reforms, including workforce strategies, governance changes, and aged care policy settings.

## 5 Conclusion

The RHMT aged care pilot programmes demonstrate that nursing and allied health placements can be established when supported by strong academic–provider partnerships, positive early experiences, and context‐sensitive implementation. The perceived benefits contributed to early local engagement and helped seed a culture of learning within services.

At the same time, the structural challenges identified highlight the need for adaptability in implementation alongside sustained system‐level support. As the RHMT aged care programme expands, continued investment in partnership structures, clinical education roles, and flexible design will be essential, supported by further research examining the educational and service impacts of placements.

## Author Contributions

**Myles Clarkson Fletcher:** conceptualization, writing – review and editing, writing – original draft, methodology, formal analysis, funding acquisition, validation, data curation. **Kathryn Fitzgerald:** conceptualization, editing and writing, methodology, formal analysis, validation, funding acquisition, data curation. **Gordon Mander:** project management, funding acquisition, reporting and coordinating, data acquisition, reviewing and editing, data management. **Pascale Dettwiller:** writing – review and editing, methodology, data curation. **Mohammad Hamiduzzaman:** conceptualization, writing – review and editing, methodology, validation, formal analysis. **Lisa Dalton:** validation, formal analysis, supervision.

## Funding

The project was funded through the Australian Rural Health Education Network (ARHEN) and participating University Departments of Rural Health (UDRHs).

## Ethics Statement

An initial low risk application was submitted to the University of Queensland and approved (Approval ID 2023/HE002384). This was then ratified and approved by the University of Western Australia (Approval ID 2024/ET000126). Subsequent ethics submissions and approvals were made to the University of Tasmania (Approval ID H30133), and the University of South Australia (Approval ID 206218). The University of Sydney registered the initial University of Queensland ethics application. All UDRHs involved in the research were contacted to ensure a mutual understanding of the project's purpose and their involvement.

## Conflicts of Interest

The authors declare no conflicts of interest.

## Data Availability

The data that support the findings of this study are available from the corresponding author upon reasonable request.

## Endnotes

^1^
Thematic saturation was assessed retrospectively using the ‘new information threshold’ method (4): • base size ‐ the minimum number of interviews to use as the denominator representing the amount of information already gained • run length – the number of consecutive interviews within which to calculate new information (the numerator) • new information threshold – the level of paucity of new information acceptable as indicative of saturation (≤ 5% is recommended).
